# Contribution of Nonaxial n → σ* Orbital Interactions in Tetrel Bonding: A Case Study on Si…N Interactions

**DOI:** 10.1002/cphc.202500042

**Published:** 2025-07-16

**Authors:** Anjali Devi Vasarla, Anik Sen, Rahul Shukla

**Affiliations:** ^1^ CMDD Laboratory Department of Chemistry School of Science GITAM (Deemed to be University) Visakhapatnam Andhra Pradesh 530045 India; ^2^ NCI Laboratory Department of Chemistry School of Science GITAM (Deemed to be University) Visakhapatnam Andhra Pradesh 530045 India

**Keywords:** σ‐hole, density functional theory, noncovalent interactions, orbital interactions, tetrel bonding

## Abstract

The formation of a *σ*‐hole interaction is typically associated with a corresponding *n* → σ* orbital interaction. This study examines the role of these *n* → σ* interactions in stabilizing Si…N tetrel bonding within various dimeric complexes. While conventional *n* → σ* orbital overlap along the tetrel bonding axis is observed as expected, the findings highlight the presence and significant contribution of additional nonaxial *n* → σ* orbital interactions in *sp* ^3^‐hybridized silicon dimers. Traditional theories of tetrel bonding emphasize linear interactions along the *σ*‐hole axis, characterized by direct head‐to‐head *n* → σ* overlaps. However, this study reveals that nonaxially oriented σ* orbitals of the tetrel atom can also substantially interact with electron donors, significantly enhancing the stability of Si…N tetrel bonds. Depending on the substituents in the dimeric complexes, nonaxial *n* → σ* interactions can contribute notably higher to the stabilization than their axial counterparts.

## Graphical abstract

This study reveals the critical role of nonaxial n → σ* orbital interactions in stabilizing Si···N tetrel bonds. Through computational analysis and comparison with crystal structures, it is demonstrated that nonaxial interactions significantly enhance bond strength and complement conventional axial bonding, offering new insights into the nature of σ‐hole interactions.

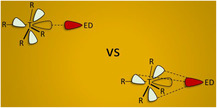

## 1 Introduction

When isolated atoms with uniform electron density come together to form a molecule, the electron density redistributes in an anisotropic manner.^[^ ^1^ ^]^ This anisotropic redistribution of electron density creates regions of relatively high electron density and relatively low electron density throughout the molecule. When these regions of relatively low electron density, also known as a “hole”,^[^ ^2^, ^3^ ^]^ are present on an atom along the extension of a *σ*‐covalent bond, they are termed a *σ*‐hole.^[^ ^4^ ^]^ A region of positive potential often characterizes the presence of a *σ*‐hole on an atom on the electrostatic potential (ESP) map.^[^ ^2^ ^]^

**Figure 1:**
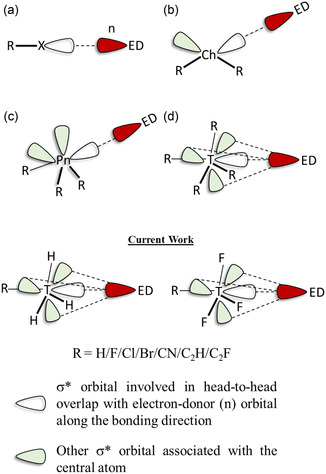
Schematic representation of different σ* orbitals in (a) halogen bonding (b) chalcogen bonding (c) pnictogen bonding (d) tetrel bonding, and the detail of Si···N tetrel bonding interaction investigated in this current study.

A *σ*‐hole interaction is the attractive noncovalent interaction between the *σ*‐hole and an electron‐donating region, present on the same or a different molecule.^[^ ^4^ ^]^ Noncovalent interactions such as halogen bonding,^[^ ^5^, ^6^ ^]^ chalcogen bonding,^[^ ^7^, ^8^ ^]^ pnictogen bonding,^[^ ^9^, ^10^ ^]^ and tetrel bonding^[^ ^11^, ^12^ ^]^ can also be categorized as *σ*‐hole interactions (**Figure** **1**). From a molecular‐orbital perspective,^[^ ^13^, ^14^, ^15^, ^16^, ^17^, ^18^ ^]^ the formation of the *σ*‐hole interaction is also described as the *n* → σ* interactions involving the anti‐bonding orbital (σ*) of the electron‐acceptor and electron‐donor orbital (n). It is also important to note that *σ*‐hole interactions are usually considered directional interactions. This directionality arises because the *σ*‐hole region usually aligns with another molecule's electron‐donating atom or group. This alignment often results in a close to linear geometry, which minimizes repulsion and optimizes the overlap between the electron‐deficient and electron‐rich regions. In the case of halogens, only one hole region and the associated σ* orbital interact with the electron‐donor (Figure 1a). On the other hand, chalcogens and pnictogens have multiple *σ*‐hole regions and associated σ* orbitals. However, the geometry of the chalcogen (Figure 1b) and pnictogen bonding complexes (Figure 1c) allows only one σ* orbital to efficiently participate in the interaction with a given electron donor (ED) region. In other words, a single ED orbital can interact with only one σ* orbital at a time.

In the case of a *sp* ^3^‐hybridized tetrel atom, the orientation of the *σ*‐hole region and associated σ* orbitals relative to the electron donor differs significantly (Figure 1d). Among the four possible *σ*‐hole regions associated with the tetrel atom, only one aligns linearly with the electron‐donor along the primary bonding axis. This linear alignment positions the *σ*‐hole directly along the axial bonding direction, facilitating a conventional, direct *σ*‐hole interaction accompanied by *n* → σ* orbital interaction. However, the remaining three *σ*‐hole regions, are oriented nonlinearly and positioned off‐axis relative to the bonding direction (Figure 1d). The σ* orbitals associated with these nonaxial regions can also possibly interact with the electron donor orbitals, albeit nonlinearly. Additionally, in molecules with tetrel atoms other than carbon, the ideal tetrahedral geometry often gets distorted upon the formation of tetrel bonding.^[^ ^19^ ^]^ This distortion can increase the likelihood of interactions between the nonaxial σ* orbital regions and the electron donor orbitals. Despite their potential importance, nonaxial interactions are often overlooked in tetrel bonding studies, which typically emphasize linear, axial σ‐hole interactions. Therefore, while many studies have been reported for the tetrel bonding interactions in the past few years,^[^ ^11^, ^12^, ^19^, ^20^, ^21^, ^22^, ^23^, ^24^, ^25^, ^26^ ^]^ a detailed analysis of nonaxial *n* → σ* orbital interaction has not been focused upon previously. This study addresses this gap by quantitively investigating the contribution of nonaxial *n* → σ* orbital interaction in tetrel bonding interactions. The contributions of these off‐axis σ* orbitals, associated with the three other regions in a tetrahedral geometry have been largely neglected, even though they may influence the bond's strength, geometry, and properties. By examining how these nonlinear orbital interactions affect the overall strength and behavior of the tetrel bond, this study offers fresh insights into the role of multidirectional interactions in tetrel bonding. This work fills a critical gap by redefining the scope of tetrel bonding to include nonlinear contributions, which could be key in designing and predicting new molecular structures involving tetrel atoms.

Therefore, through comprehensive geometrical, energetic, electron‐density topology, and most importantly through molecular orbital analyses of R‐H_3_Si⋅⋅⋅NH_3_ and R‐F_3_Si⋅⋅⋅NH_3_ (R = H/F/Cl/Br/CN/C_2_H/C_2_F), we explored the effect of axial substitution (R) on the nature and properties of Si⋅⋅⋅N tetrel bonding (Figure 1). Additionally, and more importantly, through fluorination, we explored how orbital interaction occurring between the nonaxial σ* orbital and the nitrogen lone pair contribute towards the strength and stability of the Si⋅⋅⋅N tetrel bond.

## 2 Computational Details

All computations in this study were carried out at the M06‐2X/aug‐cc‐pVDZ level of theory, which is known to provide reliable results for the noncovalent interactions.^[^ ^27^, ^28^, ^29^, ^30^ ^]^ Geometry optimization and vibrational analysis for both monomers and dimers were conducted using the Gaussian16 software package.^[^ ^31^ ^]^ The absence of any imaginary frequencies was confirmed to ensure that the optimized structures correspond to energy minima. The interaction energy (*E*_IE_) of the dimers was calculated as the difference between the energy of the optimized dimer and the sum of the energies of the monomers (*E*_A_ and *E*_B_) at the complex geometry. For comparison, interaction energy (E_IE_) was also computed at the M06‐2X/aug‐cc‐pVQZ level utilizing the optimized geometries obtained at M06‐2X/aug‐cc‐pVDZ level. Further, the effect of basis set superimposition error (BSSE) on *E*_IE_ was computed at both M06‐2X/aug‐cc‐pVDZ and M06‐2X/aug‐cc‐pVQZ level. The binding energy (*E*_BE_) was determined as the difference between the energy of the optimized dimer and the sum of the energies of the monomers (*E*_A_ and *E*_B_) at their individually optimized geometries. The deformation energy (*E*_DE_) was obtained by subtracting *E*_IE_ from *E*_BE_. Topological analysis of electron density, based on the Quantum Theory of Atoms in Molecules (QTAIM),^[^ ^32^ ^]^ was performed to extract parameters such as electron density (*ρ*) and the Laplacian (∇^2^ *ρ*) and total energy density (H) at the bond critical points. Second‐order perturbation energy (E^2^) was calculated using the NBO7^[^ ^33^ ^]^ software to explore the orbital contributions to the noncovalent interactions. For deeper insights into the energetics, localized molecular orbital energy decomposition analysis (LMO‐EDA) was performed for all dimeric dimers using the GAMESS(US) software package.^[^ ^34^ ^]^

## 3 Results and Discussion

### 3.1 Geometry of the Monomers

**Figure 2:**
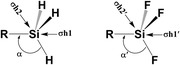
Important bond angle and unique σ‐hole (σh) regions present in the R‐H_3_Si and R‐F_3_Si monomers.

The analysis of the geometry of R‐H_3_Si monomers (where R = H, F, Cl, Br, CN, C_2_H, C_2_F) reveals that the ∠R–Si–H (α_m_ ; m = monomer) (**Figure** **2**, **Table** **1**) varies between 107.0° (for R = CN) and 109.5° (for R = H). In the corresponding R‐F_3_Si monomers, the ∠R–Si–F (α′_m_) ranges from 109.5° (for R = F) to 111.3° (for R = H or C_2_F). Notably, the magnitude of α′_m_ is consistently larger than that of α_m_ in the corresponding monomers; this is due to fluorine's high electronegativity and greater steric effects, which lead to electron withdrawal and angle expansion. This structural trend reflects a preference for larger bond angles in fluorine‐substituted silanes.

**Table 1:** The magnitude of the important bond angle and the magnitude of the ESP at the σ‐hole region in the monomers.

	R‐H_3_Si			R‐F_3_Si		
R	α_m_ [°]	*σ*h1 [kJ mol^−1^]	*σ*h2 [kJ mol^−1^]	α′_m_ [°]	*σ*h1′ [kJ mol^−1^]	*σ*h2′ [kJ mol^−1^]
H	109.5	69.9	69.9	111.3	134.9	181.4
F	107.6	157.9	96.9	109.5	204.8	204.8
Cl	107.7	147.0	84.6	110.6	170.1	161.9
Br	107.6	145.2	81.5	110.9	165.4	149.7
CN	107.0	167.4	121.5	109.6	219.4	210.9
C_2_H	108.9	98.1	62.6	111.2	133.2	143.2
C_2_F	108.9	102.6	67.1	111.3	134.8	143.3

### 3.2 ESP Map

**Figure 3:**
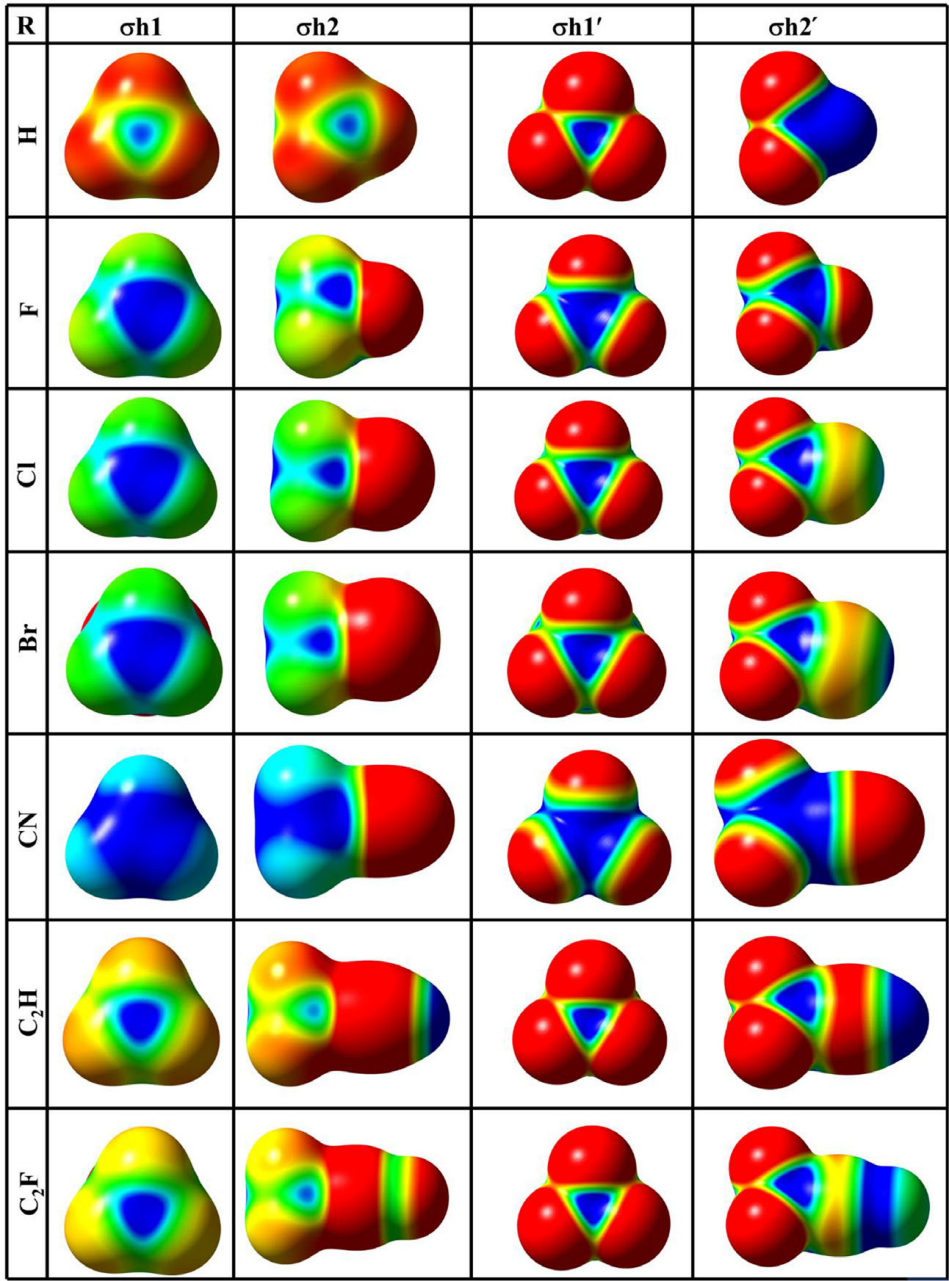
ESP map of R‐H_3_Si monomers (along *σ*h1 and *σ*h2) and R‐F_3_Si monomers (along *σ*h1´ and *σ*h2´) drawn at 0.001 au electron‐density isosurface with color ranging from red (−75 kJ mol^−1^) to blue (75 kJ mol^−1^).

ESP mapping further reveals two distinct *σ*‐hole regions on the silicon atom in each monomer: one aligned with the extension of the R–Si bond (*σ*h1 or *σ*h1′) and another along each of the H–Si bond (*σ*h2) in R‐H_3_Si monomers, or along the F–Si bond (*σ*h2′) in R‐F_3_Si monomers (**Figure** 2 and **3**). The magnitude of the ESP at *σ*h1 region is well correlated with the electron‐withdrawing nature of the axial substituent R. The magnitude of ESP at *σ*h1 ranged from 69.9 kJ mol^−1^ (R = H) to 167.4 kJ/mol (R = CN) (Table 1). On the other hand, the magnitude of ESP at *σ*h2 ranged from 62.6 kJ/mol (R = C_2_H) to 121.5 kJ mol^−1^ (R = CN). This means that in all the R‐H_3_Si monomers, *σ*h1 region appears to be more electron‐deficient than *σ*h2 (expect for R = H, where *σ*h1 = *σ*h2). Once hydrogen is replaced by fluorine, resulting in R‐F_3_Si monomers, both the *σ*‐hole regions (*σ*h1′ and *σ*h2′) become more electron‐deficient as indicated by the higher magnitude of ESP at these regions. More interestingly, in R‐F_3_Si monomers, the ESP at the *σ*h1′ is either equal (as in SiF_4_), greater (as in SiF_3_Cl, SiF_3_Br, and SiF_3_CN), or even lower (as in SiF_3_H, SiF_3_C_2_H, and SiF_3_C_2_F) than that at the *σ*h2′ (Table 1). This variation is influenced by the combined electron‐withdrawing effects of both the R substituent and the fluorine atoms within the molecule. The electron‐withdrawing power of the R group impacts the electron density distribution around the silicon center, affecting the ESP at the R–Si σ‐hole. Simultaneously, the presence of multiple fluorine atoms, which are highly electronegative, intensifies the electron deficiency at both σ‐holes on silicon. These effects modulate the relative ESP magnitudes at the R–Si and F–Si σ‐holes across different R‐F_3_Si structures.

### 3.3 Geometry of the Dimers

**Figure 4:**
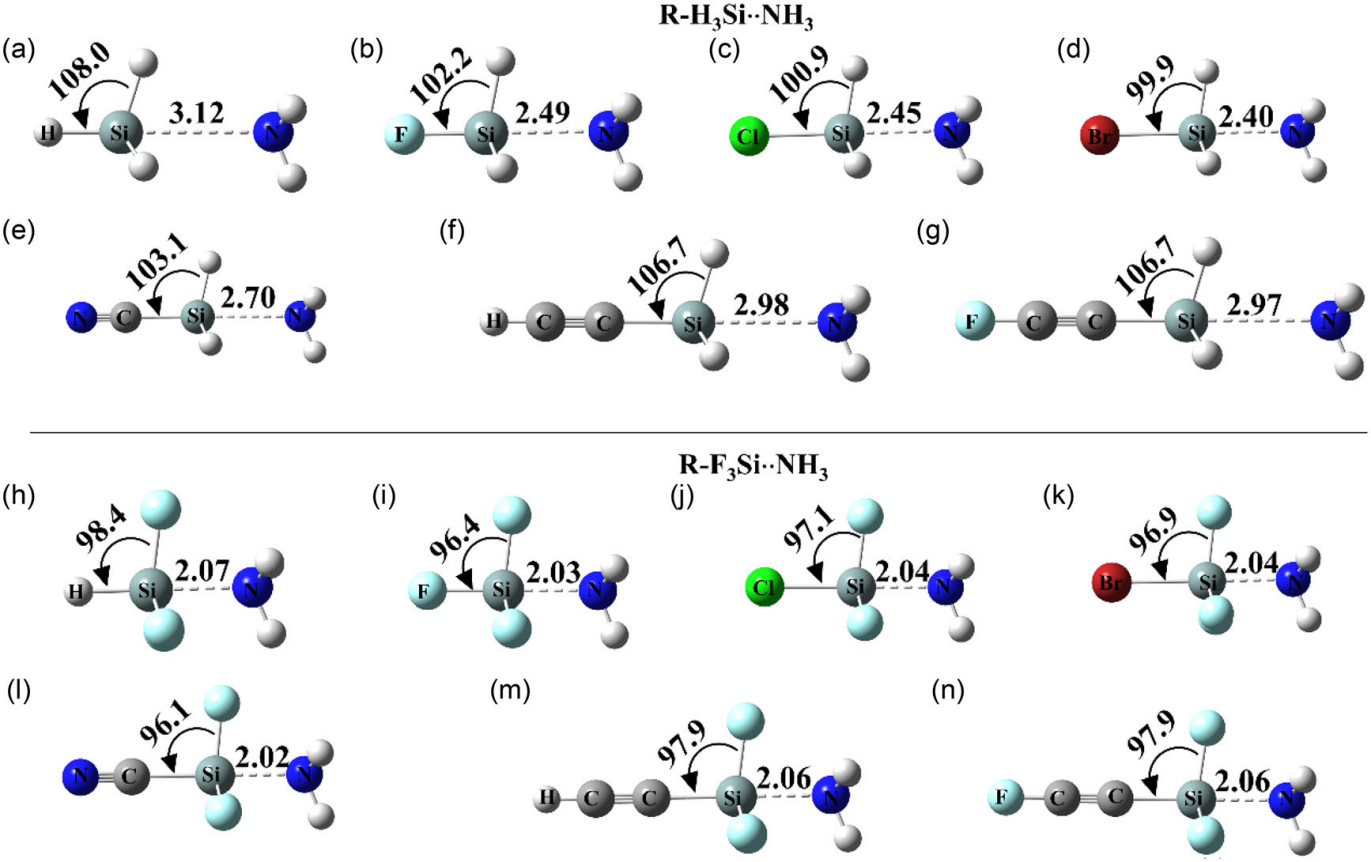
Molecule structure of the a–g) R‐H_3_Si···NH_3_ dimers along with Si···N bond distances (in Å) and α_d_ (in °). Molecule structure of the h–n) R‐F_3_Si···NH_3_ dimers along with Si···N bond distances (in Å) and α´_d_ (in °).

Next, we optimized all the monomers with NH_3_ along the *σ*h1 or *σ*h1′ region, resulting in the formation of R‐H_3_Si···NH_3_ and R‐F_3_Si···NH_3_ dimers stabilized by directional R‐Si···N tetrel bonding interaction (**Figure** **4**). All interactions were linear directional interactions with ∠R‐Si···N being 180° in all the dimers. For the R‐H_3_Si···NH_3_ complex, Si···N distance ranged from 2.40 Å (R = Br) to 3.12 Å (R = H) (**Table** **2**). The magnitude of the reduction ratio (RR), defined as the ratio of the distance of the noncovalent interaction and sum of the vdW radii of the interacting atoms, i.e., $\frac{d\left(SiN\right)}{vdW\left(Si+N\right)}$, ranged from 0.66 (R = Br) to 0.85 (R = H). The Si···N bond distance was shorter for the highly electronegative halogen substituents (R = F/Cl/Br) than for other substituents. It is also important to note that shorter Si···N also led to a smaller magnitude of **α**_**d**_ (∠R‐Si‐H in the dimer) as compared to the magnitude of α_m_ observed in the corresponding monomers, resulting in the negative magnitude of Δ*α* (Δ*α* = α_d_ − α_m_), ranging from −1.5° (R = H) to −7.7° (R = Br). Again, this angular deformation is larger for R = F/Cl/Br than for other substituents. Overall, all the geometrical variation observed in the case of the R‐H_3_Si···NH_3_ dimers is highly dependent on the electronic behavior of the substituent R.

**Table 2:** Important geometrical parameters associated with R‐H_3_Si···NH_3_ and R‐F_3_Si···NH_3_ dimers.

	R‐H_3_Si···NH_3_				R‐F_3_Si···NH_3_			
R	Si···N	RR	α_d_	Δα	Si···N	RR	α′_d_	Δα′_d_
H	3.12	0.85	108.0	− 1.5	2.07	0.57	98.4	− 12.9
F	2.49	0.68	102.2	− 5.4	2.03	0.56	96.4	− 13.1
Cl	2.45	0.67	100.9	− 6.8	2.04	0.56	97.1	− 13.5
Br	2.40	0.66	99.9	− 7.7	2.04	0.56	96.9	− 14.0
CN	2.70	0.74	103.1	− 3.9	2.02	0.55	96.1	− 13.5
C_2_H	2.98	0.82	106.7	− 2.2	2.06	0.56	97.9	− 13.3
C_2_F	2.97	0.81	106.7	− 2.2	2.06	0.56	97.9	− 13.4

In comparison, the variation in Si···N distance in R‐F_3_Si···NH_3_ dimers was very narrow as the distance ranged from 2.02 Å (R = CN) to 2.07 Å (R = H) (Table 2, Figure 4). This narrow variation also resulted in all the dimers having a similar RR value of ≈0. 56. The magnitude of α′_d_ ranged from 96.1 (R = CN) to 98.4 (R = H), suggesting that R‐F_3_Si···NH_3_ dimers adopt a geometry closer to a trigonal bipyramidal and therefore significantly distort the tetrahedral geometry of the R‐F_3_Si molecules. The angular deformation (Δ*α*′) while being larger than the corresponding magnitude of Δα, had a narrow range of −12.9° (R = H) to −14.0° (R = Br). Overall, in the case of R‐F_3_Si···NH_3_ dimers, the effect of the substituent R seems to be diminished as the geometry of all the dimers is similar to each other. This shows that the fluorine atoms at the nonaxial positions dominate the geometry, minimizing the influence of substituent R on the complex geometry and potentially affecting the interaction stability and electronic properties of the system.

### 3.4 Energies of the Dimers

The geometries of the dimers align well with the interaction and binding energies calculated for the dimeric dimers. In the R‐H_3_Si···NH_3_ dimers, the interaction energies (E_IE_) ranged from −13.0 kJ mol^−1^ (for R = H) to −48.7 kJ mol^−1^ (for R = Br) (**Table** **3**). Conversely, the binding energies for these dimers (E_BE_) spanned from −12.3 kJ mol^−1^ (R = H) to −32.6 kJ mol^−1^ (R = F) (Table 3). For most substituents except R = H, C_2_H, and C_2_F, there is a notable difference between the interaction and binding energies, suggesting the deformation of the R‐H_3_Si monomers upon complexation. The deformation energy (E_DE_ = E_BE_ – E_IE_), therefore, ranges from 0.7 kJ mol^−1^ (R = H) to 16.4 kJ mol^−1^ (R = Br), with higher deformation energy values indicating deviation from the tetrahedral geometry of R‐H_3_Si monomers. For the R‐F_3_Si···NH_3_ dimers, the magnitude of both the interaction and binding energies are significantly larger than those observed in the R‐H_3_Si···NH_3_ dimers. The interaction energies range from −138.7 kJ mol^−1^ (R = H) to −180.5 kJ mol^−1^ (R = CN), while binding energies vary from −46.0 kJ mol^−1^ (R = H) to −85.2 kJ mol^−1^ (R = CN). Notably, the deformation energies are substantial, with values from 83.8 kJ mol^−1^ (R = C_2_H) to 100.0 kJ mol^−1^ (R = Br) (Table 3). These high deformation energy values highlight that the presence of fluorine at nonaxial positions induces significant structural distortion in the R‐F_3_Si monomers, indicating substantial deviation from their typical geometry upon complex formation.

**Table 3:** Magnitude of the different energies associated with the Si···N interaction in R‐H_3_Si···NH_3_ and R‐F_3_Si···NH_3_ dimers. All values in kJ/mol.

R	***E***_IE_	*E*_BE_	*E*_DE_	*E*_elec_	*E*_pol_	*E*_ex‐rep_	*E*_disp_	*E*_total_
R‐H_3_Si···NH_3_ dimers								
H	− 13.0	− 12.3	0.7	− 22.7 *(41%)*	− 13.1 *(23%)*	42.6	− 20.2 *(36%)*	− 13.3
F	− 41.8	− 32.6	9.2	− 106.9 *(51%)*	− 59.5 *(28%)*	167.2	− 44.6 *(21%)*	− 43.7
Cl	− 44.8	− 31.5	13.3	− 122.9 *(51%)*	− 71.0 *(30%)*	193.3	− 46.4 *(19%)*	− 46.9
Br	− 48.7	− 32.3	16.4	− 138.2 *(51%)*	− 82.3 *(31%)*	217.4	− 48.4 *(18%)*	− 51.4
CN	− 34.5	− 29.9	4.6	− 70.2 *(50%)*	− 35.1 *(25%)*	103.4	− 33.8 *(24%)*	− 35.7
C_2_H	− 20.0	− 18.5	1.5	− 34.1 *(45%)*	− 17.5 *(23%)*	56.0	− 24.8 *(32%)*	− 20.5
C_2_F	− 20.7	− 19.1	1.6	− 35.4 *(45%)*	− 17.7 *(22%)*	57.4	− 25.6 *(33%)*	− 21.2
R‐F_3_Si···NH_3_ dimers								
H	− 138.7	− 46.0	92.7	− 324.7 *(54%)*	− 183.8 *(31%)*	460.3	− 92.0 *(15%)*	− 140.2
F	− 166.4	− 77.1	89.2	− 352.6 *(54%)*	− 207.1 *(32%)*	485.9	− 94.2 *(14%)*	− 168.0
Cl	− 163.3	− 66.2	97.1	− 348.5 *(54%)*	− 205.3 *(32%)*	482.5	− 93.9 *(14%)*	− 165.1
Br	− 165.7	− 65.7	100.0	− 350.4 *(54%)*	− 207.4 *(32%)*	483.7	− 93.7 *(14%)*	167.8
CN	− 180.5	− 85.2	95.3	− 364.0 *(54%)*	− 218.7 *(32%)*	495.1	− 94.7 *(14%)*	− 182.3
C_2_H	− 147.6	− 51.4	83.8	− 335.3 *(54%)*	− 193.3 *(31%)*	472.5	− 93.1 *(15%)*	− 149.2
C_2_F	− 148.2	− 51.4	96.8	− 335.5 *(54%)*	− 192.8 *(31%)*	472.4	− 93.7 *(15%)*	− 149.6

For comparison, we also computed the *E*_IE_ at the M06‐2X/aug‐cc‐pVQZ level of theory (Table S1, Supporting Information). The interaction energies were observed to be slightly less negative than those observed for the M06‐2X/aug‐cc‐pVDZ level. However, the trend in the energy remained the same. Further, the effect of the basis‐set superimposition error (BSSE) correction was observed to be more pronounced for the aug‐cc‐pVDZ basis set as compared to the aug‐cc‐pVQZ basis set (Table S1, Supporting Information). Despite this, the qualitative agreement between the aug‐cc‐pVDZ and aug‐cc‐pVQZ level results confirms that the uncorrected aug‐cc‐pVDZ interaction energies reliably capture the relative strength of interactions across the series, making it a suitable and cost‐effective choice for trend analysis in this study.

### 3.5 Energy Decomposition Analysis

To gain a deeper understanding of the energetics governing the Si···N interactions, we performed a LMO‐EDA. This analysis separates the total interaction energy (*E*_total_) of each complex into electrostatic (*E*_elec_), exchange‐repulsion (*E*_ex‐rep_), polarization (*E*_pol_), and dispersion energy (*E*_disp_) components (Table 3). To clarify the role of various energy components, we computed the percentage contribution of each stabilizing factor. This was determined by dividing the individual stabilizing component (*E*_elec_, *E*_pol_, or *E*_disp_) by the total stabilizing energy (*E*_elec_ + *E*_pol_ + *E*_disp_) (Table 3, *values in parenthesis*). In the R‐H_3_Si···NH_3_ dimers, stability is primarily driven by electrostatics, with the contribution ranging from 41% (R = H) to 51% (R = F/Cl/Br) towards stabilization. The contribution of polarization ranged from 22% (R = C_2_F) to 31% (R = Br) while that of dispersion ranged from 18% (R = Br) to 36% (R = H) towards stabilization. For dimers with strong electron‐withdrawing groups (R = F, Cl, Br, I), polarization contributed more significantly to stabilization than dispersion. In contrast, for the remaining dimers, dispersion played a larger role in stabilization. For the R‐F_3_Si···NH_3_ dimers, the interaction is even shorter and stronger, leading to an increased electrostatic + polarization contribution towards stabilization, ranging from 85 to 86% across all substituents R (Table 3). The contribution of dispersion was ≈15% for all the dimers. Overall, the energy decomposition was similar for all seven dimers, irrespective of the substituent R. This further suggests that the stability of the R‐F_3_Si···NH_3_ dimers is primarily influenced by the presence of fluorine in nonaxial positions, rather than by the nature of the axial substituent R.

### 3.6 Topological Analysis

We also conducted QTAIM analysis, which further supported the findings from other analyses. The identification of a bond critical point (bcp) between the interacting silicon and nitrogen atoms confirmed the presence of the Si···N tetrel bonding interaction (Figure S1, Supporting Information). As anticipated, the topological parameters, electron density (*ρ*) and Laplacian (∇^2^ *ρ*) at the Si···N bcp were significantly higher for the R‐F_3_Si···NH_3_ dimers compared to the R‐H_3_Si···NH_3_ dimers, indicating notable differences between the two sets (**Table** **4**). Additionally, both sets of dimers showed a negative total energy density (H) at the Si···N bcp (except for R = H,C_2_H, C_2_F in R‐H_3_Si···NH_3_), suggesting a partial covalent character in this interaction. Importantly, the total energy density (H) was substantially more negative in the R‐F_3_Si···NH_3_ dimers (Table 4), indicating that the Si···N interaction in these dimers has a significantly greater covalent character, consistent with the shorter and stronger nature of the Si···N interaction in the R‐F_3_Si···NH_3_ dimers.

**Table 4:** Topological parameters associated with Si···N tetrel bond.

R	R‐H_3_Si···NH_3_			R‐F_3_Si···NH_3_		
	*ρ* [e/Å^3^]	*∇* ^2^ *ρ* [e/Å^5^]	H [Hartree/Å^3^]	*ρ* [e/Å^3^]	*∇* ^2^ *ρ* [e/Å^5^]	H [Hartree/Å^3^]
H	0.069	0.617	0.003	0.400	4.036	− 0.124
F	0.194	1.108	− 0.040	0.436	4.832	− 0.132
Cl	0.215	1.054	− 0.052	0.428	4.630	− 0.131
Br	0.234	1.059	− 0.064	0.430	4.669	− 0.131
CN	0.136	0.945	− 0.012	0.447	5.112	− 0.133
C_2_H	0.085	0.750	0.001	0.413	4.312	− 0.128
C_2_F	0.086	0.764	0.001	0.413	4.309	− 0.128

### 3.7 Bonding Orbital Analysis

Based on the analysis performed so far, it is evident that even nonaxial substitution can influence the geometry, energies, and topology of the Si···N tetrel bond. In the case of R‐F_3_Si···NH_3_ dimers, the presence of fluorine in the nonaxial position significantly overshadows the effect of the axial substituent (R). As a result, all the dimers exhibit similar characteristics, regardless of the substituent R. In these dimers, the C—F bond forms a trigonal plane (Figure 4), which likely allows the three nonaxial Si‐F σ* orbitals to strongly overlap with the nitrogen lone pairs, thus enabling a nonaxial *n* → σ* overlap. To test this theory, we performed the natural bond orbital (NBO) analysis to obtain the second‐order perturbation energy (E^2^) for all the N(*lp*) → σ*(Si‐R/H/F) orbital interactions occurring in the R‐H_3_Si···NH_3_ and R‐F_3_Si···NH_3_ dimers.

For all dimers, an axial N(*lp*) → σ*(Si‐R) orbital interaction exists, which aligns with expectations for *σ*‐hole interactions where a lone pair on the nitrogen (N) interacts with an electron‐deficient region (σ*) associated with silicon along the bonding direction. However, beyond this primary axial interaction, both dimers display an additional set of three equivalent nonaxial interactions. Specifically, in the R‐H_3_Si···NH_3_ dimers, these interactions are represented by N(*lp*) → σ*(Si‐H), whereas in R‐F_3_Si···NH_3_, they are N(*lp*) → σ*(Si‐F) interactions. This shows that the nitrogen atom's lone pair does not only interact with the axial σ‐hole region on Si but also engages with three nonaxial σ‐hole regions. The second‐order perturbation energies (E^2^), as displayed in **Table** **5**, quantify these interactions, offering a measure of their stabilizing effects. It is important to note that the reported values for the N → σ* (Si‐H) or N → σ* (Si‐F) nonaxial interactions are the sum of the three equivalent contributions.

**Table 5:** The magnitude of E^2^ associated with N → σ* orbital interaction in all the Si···N interactions investigated in the study. All values in kJ mol^−1^.

	R‐H_3_Si···NH_3_			R‐F_3_Si···NH_3_		
R	N(*lp*) → σ*(Si‐R) (axial)	N(*lp*) → σ*(Si‐H)a) *(nonaxial)*	Total	N(*lp*) → σ*(Si‐R) (axial)	N(*lp*) → σ*(Si‐F)a) *(nonaxial)*	Total
H	16.4	8.9	25.3	48.0	319.8	367.8
F	82.2	40.0	122.2	103.0	269.0	372.0
Cl	96.7	54.6	151.3	83.3	306.0	389.3
Br	110.1	64.1	174.2	80.8	315.0	395.8
CN	51.1	26.2	77.3	81.9	330.5	412.4
C_2_H	28.3	11.5	39.8	70.9	305.8	376.7
C_2_F	29.1	11.7	40.8	71.4	306.6	378.0

a) Sum of all three equivalent N(lp) → σ*(Si‐H) or N(*lp*) → σ*(Si‐F) interaction.

In the case of R‐H_3_Si···NH_3_ dimers, the E^2^ value for the axial N(*lp*) → σ*(Si‐R) interaction ranged from 16.4 kJ mol^−1^ (R = H) to 110.1 kJ mol^−1^ (R = Br) (Table 5). In comparison, the cumulative contribution of N(*lp*) → σ*(Si‐H) ranged from 8.9 kJ mol^−1^ (R = H) to 64.1 kJ mol^−1^ (R = Br). Therefore, for R‐H_3_Si···NH_3_ dimers, the axial N(*lp*) → σ*(Si‐R) interaction is always dominant over the nonaxial N(*lp*) → σ*(Si‐H) orbital interaction. However, the nonaxial N(*lp*) → σ*(Si‐H) contribution is also substantial and should not be neglected. On the other hand, in the case of R‐F_3_Si···NH_3_ dimers, the E^2^ value for the axial N(*lp*) → σ*(Si‐R) interaction ranged from 48.0 kJ mol^−1^ (R = H) to 103.0 kJ mol^−1^ (R = F). In comparison, the cumulative contribution of N(*lp*) → σ*(Si‐F) ranged substantially higher, from 269.0 kJ mol^−1^ (R = F) to 330.5 kJ mol^−1^ (R = CN). These results indicate that in R‐F_3_Si···NH_3_ dimers, the nonaxial N(*lp*) → σ*(Si‐F) interaction plays a much more significant role than the axial N(*lp*) → σ*(Si‐R) interaction. This shows the involvement of the nonaxial‐hole regions in forming short and stable Si···N interactions. Overall, this highlights the importance of considering both axial and nonaxial interactions in these systems.

**Figure 5:**
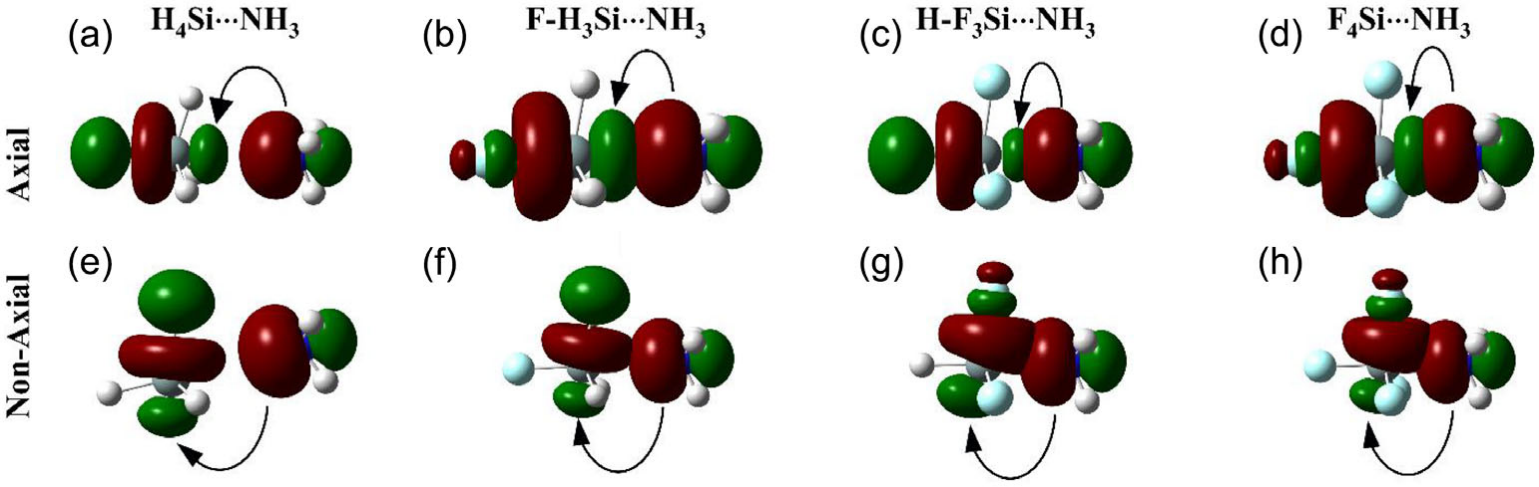
Representation of (a)‐(d) axial and (e)‐(h) nonaxial N(lp) → σ* orbital interaction in Si···N tetrel bonding interaction.

To further analyze, we also plotted the axial and nonaxial orbitals for selected dimers, namely H_4_Si···NH_3_, FH_3_Si···NH_3_, HF_3_Si···NH_3_, and F_4_Si···NH_3_. **Figure** **5**a–d represents the linear orbital interaction occurring between the N(*lp*) orbital and the axial σ* orbital lying along the Si···N bonding axis. On the other hand, 5(e)‐(h) represents one of the nonlinear orbital interactions occurring between the N(*lp*) orbital and the nonaxial σ* orbital. The NBO analysis shows that while the N(*lp*) orbital and the nonaxial σ* orbital are roughly orthogonal, the charge transfer between these orbitals occurs. The extent of the orbital interaction is highly dependent on the nature of the substituent on the nonaxial position as it determines the depth of the σ‐hole present.

### 3.8 Crystallographic Evidence

**Figure 6:**
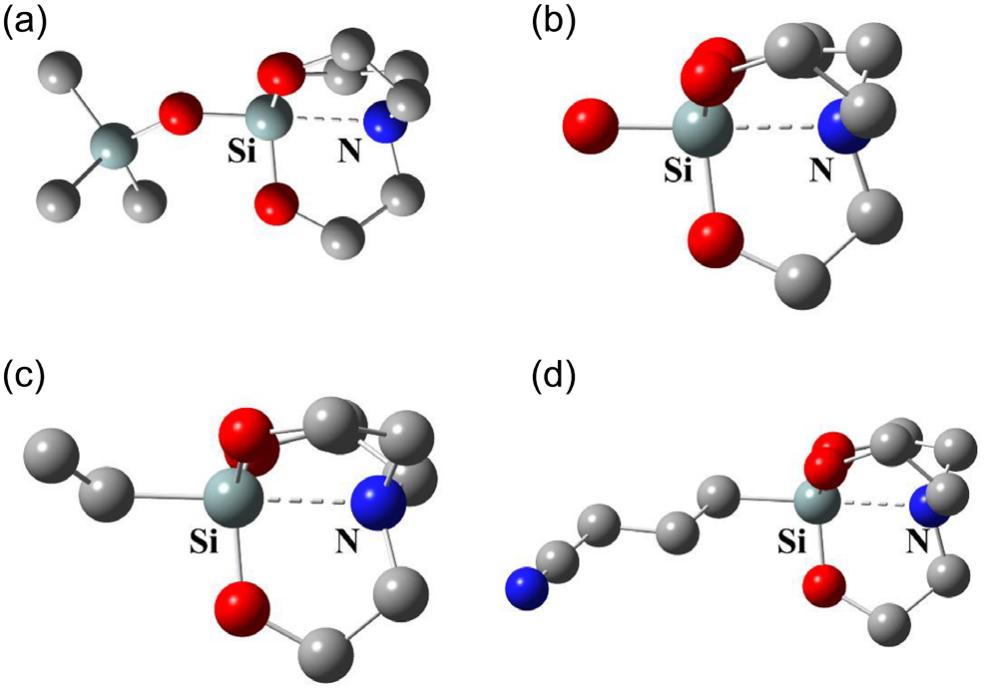
Molecular structure of the four crystal structures analyzed in the study (a) AZASAV (b) AZARUO (c) BACGAN02 (d) BAZCEK10. Hydrogens are removed for clarity.

Our theoretical study on the model systems R‐H_3_Si···NH_3_ and R‐F_3_Si···NH_3_ dimers highlights the involvement of the nonaxial σ‐hole region in Si···N tetrel bonding interactions. To corroborate these findings, we further examined Si···N interactions in several previously reported crystal structures. A simple literature search revealed short Si···N interactions across a diverse range of molecules.^[^ ^35^, ^36^, ^37^, ^38^ ^]^ Based on structural similarities with our model system, we selected four silatranes crystal structures^[^ ^35^ ^]^ (CSD^[^ ^39^ ^]^ REFCODE: AZASAV, AZARUO, BACGAN02, and BAZCEK10) for analysis (**Figure** **6**). In contrast to the intermolecular tetrel bonding observed in our model systems, these crystal structures exhibited intramolecular Si···N tetrel bonding. Additionally, these structures featured slightly less electronegative oxygen in the nonaxial position, instead of the fluorine used in our models, resulting in slightly longer Si···N distances, ranging from 2.11 to 2.18 Å (**Table** **6**). Each crystal structure also had three different magnitudes of α_d_, as the substituent R was nonsymmetric. Notably, the α_d_ values observed in the crystal structures were comparable to those in the R‐F_3_Si···NH_3_ dimers (Table 2). Crucially, the cumulative contribution of the nonaxial N(*lp*) → σ*(Si‐O) interaction in these crystal structures was significantly higher, with E^2^ values ranging from 195.3 to 221.9 kJ mol^−1^, compared to the axial N(*lp*) → σ*(Si‐R) interactions, which had E^2^ values between 39.0 and 62.6 kJ mol^−1^. This establishes the importance of nonaxial σ‐hole interactions in crystal structures as well as described in our study.

**Table 6:** Geometrical parameters and the E^2^ (kJ mol^−1^) energies for the Si···N tetrel bonding interaction present in the analyzed crystal structure.

REFCODE	Si···N [Å]	α_d_ [°]	N(*lp*) → σ*(Si‐R) (axial)	N(*lp*) → σ*(Si‐O) (nonaxial)
AZASAV	2.18	95.7/97.4/97.3	59.6	195.3
AZARUO	2.11	96.8/97.7/92.7	62.6	221.9
BACGAN02	2.15	96.3/96.1/97.9	49.7	220.8
BAZCEK10	2.16	95.8/98.3/96.5	39.0	202.2

## 4 Concluding Remarks

This study delved into the molecular orbital overlap between the nonaxial σ* orbital and nitrogen lone‐pairs while focusing on Si…N interactions. While traditional research on tetrel bonding has largely concentrated on linear *σ*‐hole interactions aligned with the bonding axis between the tetrel atom and an electron donor and accompanied by a head‐to‐head *n* → σ* orbital interaction, our examination of these nonaxial interactions provides a broader perspective on tetrel bonding. This approach uncovers new insights into how such interactions impact the stability, strength, and geometry of tetrel bonding interaction.

In conclusion, we offer a comprehensive understanding of the crucial role that nonaxial n → σ* interactions play in Si···N tetrel bonding, combining both theoretical calculations and crystal structure analysis. Through our investigation of two model systems, R‐H_3_Si···NH_3_ and R‐F_3_Si···NH_3_ dimers, we demonstrated that these nonaxial n → σ* interactions significantly contribute to the stabilization of Si···N bonding. Specifically, the nonaxial regions, where the σ* is not axially aligned with the incoming electron density, still participate in the bonding interaction, resulting in shorter and stronger tetrel bonds. To validate these findings, we extended our analysis to crystal structures exhibiting intramolecular Si···N tetrel bonding. These structures shared notable similarities with our model systems but with slight variations, such as the substitution of fluorine with oxygen in the nonaxial position. This modification led to slightly longer Si···N distances, yet the dominance of nonaxial orbital interactions remained clear. Interestingly, the crystal structures showed a significantly greater contribution from nonaxial N(*lp*) → σ*(Si‐O) interactions, with energies considerably higher than those of the axial N(*lp*) → σ*(Si‐R) interactions. This empirical evidence further solidifies the theoretical insights, emphasizing the pivotal role of nonaxial interactions in stabilizing Si···N bonds.

Finally, this study also opens up discussion on the definition of the σ‐hole interactions. Previously reported studies have demonstrated that the presence of an electrostatically driven σ‐hole interaction is usually accompanied by a corresponding axial *n* → σ* orbital interaction. Does it mean that the presence of the nonaxial *n* → σ* orbital interaction also suggests the presence of a nonlinear/nonaxial σ‐hole interaction or, in other words, the nonaxial σ‐hole region of silicon is interacting with the nitrogen to form Si···N nonaxial σ‐hole interaction? If we look into the literature, there are reports where the electrostatic positive region does not need to align perfectly with the electrostatic negative region to form the noncovalent interaction.^[^ ^40^ ^]^ However, further deeper insight is required to conclude. Hence, these insights open the door for further research into the impact of nonaxial interactions in other noncovalent bonding motifs, advancing our understanding of molecular interactions and their potential applications.

## Conflict of Interest

The authors declare no conflict of interest.

## Supporting information

Supplementary Material

## Data Availability

The data that support the findings of this study are available in the supplementary material of this article.
