# Supplementary material for: Contribution of Nonaxial n → σ* Orbital Interactions in Tetrel Bonding: A Case Study on Si…N Interactions
Source: Chemphyschem. 2025 Jul 16;26(17):e202500042. doi: 10.1002/cphc.202500042 (PMC12447117; doi:10.1002/cphc.202500042)
Supplement: Supplementary file 1 — Supplementary Material [file CPHC-26-e202500042-s001.pdf]

Contribution of non-axial n→σ* orbital interactions in Tetrel Bonding: A Case Study on Si···N Interactions

Anjali Devi Vasarla,[a,b] Anik Sen*[a] and Rahul Shukla*[b]

[a] *CMDD Laboratory, Department of Chemistry, School of Science, GITAM (Deemed to be University), Visakhapatnam-530045, Andhra Pradesh, INDIA, Email: asen@gitam.edu*

[b] *NCI Laboratory, Department of Chemistry, School of Science, GITAM (Deemed to be University), Visakhapatnam-530045, Andhra Pradesh, INDIA, Email: rshukla2@gitam.edu*

S. No.		Page No.
1	**Figure S1**. Molecular Graph representing Si···N bond critical point	2
2	**Table S1. Interaction Energies at different level of theories**	2
2	Dimer Coordinates	3-5

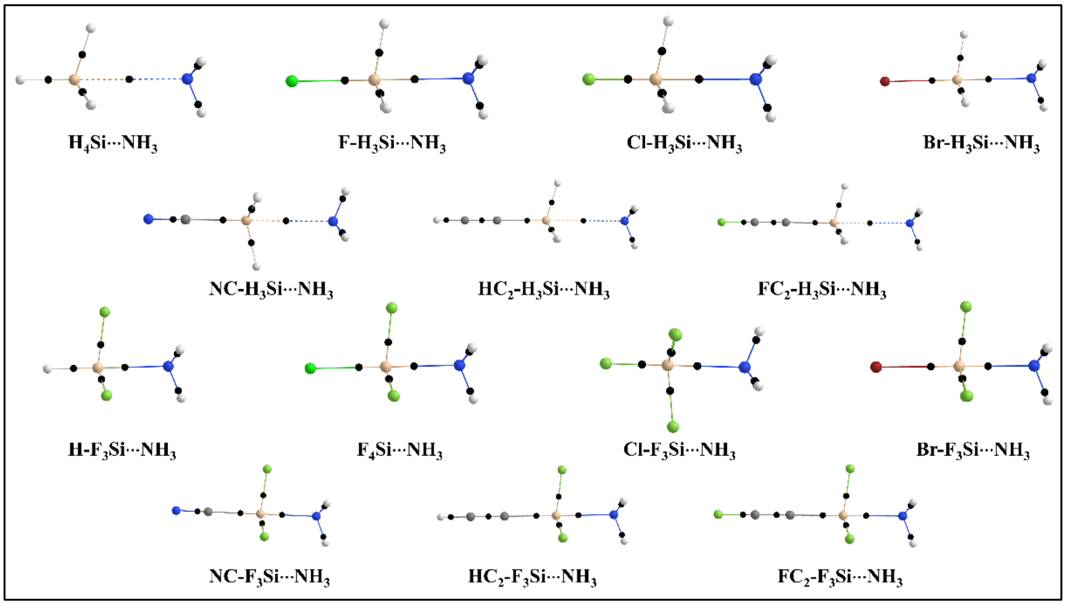

Figure S1. Molecular Graph representing Si···N bond critical point

Table S1. Interaction energies of the R-$H_3Si\cdots NH_3$ and R-$F_3Si\cdots NH_3$ dimers at different level of theories along with BSSE corrected interaction energies. All values in kJ/mol

Complex	M062X/<br>aug-cc-pVDZ	M062X/<br>aug-cc-pVDZ (BSSE)	M062X/<br>aug-cc-pVQZ	M062X/<br>aug-cc-pVQZ (BSSE)
**R-$H_3Si\cdots NH_3$ dimers**				
**H**	-13.0	-10.8	-11.4	-11.2
**F**	-41.8	-37.5	-41.4	-40.9
**Cl**	-44.8	-40.0	-43.1	-42.8
**Br**	-48.7	-43.9	-46.8	-46.4
**CN**	-34.5	-31.0	-33.4	-33.1
**C_2H**	-20.0	-17.3	-18.2	-17.9
**C_2F**	-20.7	-17.9	-18.9	-18.6
**R-$F_3Si\cdots NH_3$ dimers**				
**H**	-138.7	-129.4	-135.3	-134.1
**F**	-166.4	-155.4	-162.7	-161.2
**Cl**	-163.3	-151.2	-157.6	-156.4
**Br**	-165.7	-153.9	-160.4	-159.2
**CN**	-180.5	-169.1	-176.6	-175.3
**C_2H**	-147.6	-136.5	-142.7	-141.5
**C_2F**	-148.2	-136.9	-143.1	-141.8

Dimers Coordinates

$H_4Si{\cdots}NH_3$
Si 0.00000000 0.00000000 1.15172800
H 0.00000000 1.41343200 0.69328700
H -1.22406800 -0.70671600 0.69328700
H 1.22406800 -0.70671600 0.69328700
H 0.00000000 0.00000000 2.64711900
N 0.00000000 0.00000000 -1.97049500
H 0.00000000 -0.94214300 -2.35256900
H 0.81592000 0.47107100 -2.35256900
H -0.81592000 0.47107100 -2.35256900

$F\text{-}H_3Si{\cdots}NH_3$
Si 0.00000000 0.00000000 0.33286000
H 0.00000000 1.44822600 0.01881600
H -1.25420100 -0.72411300 0.01881600
H 1.25420100 -0.72411300 0.01881600
N 0.00000000 0.00000000 -2.16210900
H 0.00000000 -0.94492000 -2.53787300
H 0.81832500 0.47246000 -2.53787300
H -0.81832500 0.47246000 -2.53787300
F 0.00000000 0.00000000 2.00354300

$Cl\text{-}H_3Si{\cdots}NH_3$
Si 0.00000000 0.00000000 -0.23035100
H 0.00000000 1.45299500 -0.51130100
H -1.25833100 -0.72649800 -0.51130100
H 1.25833100 -0.72649800 -0.51130100
N 0.00000000 0.00000000 -2.67686300
H 0.00000000 -0.94567000 -3.05130000
H 0.81897400 0.47283500 -3.05130000
H -0.81897400 0.47283500 -3.05130000
Cl 0.00000000 0.00000000 1.92063300

$Br\text{-}H_3Si{\cdots}NH_3$
Si 0.00000000 0.00000000 -0.89621600
H 0.00000000 1.45754200 -1.15158200
H -1.26226800 -0.72877100 -1.15158200
H 1.26226800 -0.72877100 -1.15158200
N 0.00000000 0.00000000 -3.29632400
H 0.00000000 -0.94565300 -3.67175600
H 0.81895900 0.47282600 -3.67175600
H -0.81895900 0.47282600 -3.67175600
Br 0.00000000 0.00000000 1.43118000

$NC\text{-}H_3Si{\cdots}NH_3$
Si 0.00000000 0.00000000 0.09047500
H 0.00000000 1.43932100 0.42504600
H 1.24648900 -0.71966000 0.42504600
H -1.24648900 -0.71966000 0.42504600
N 0.00000000 0.00000000 2.79645300
H 0.00000000 -0.94229500 3.17968900
H -0.81605100 0.47114800 3.17968900
H 0.81605100 0.47114800 3.17968900
C 0.00000000 0.00000000 -1.81105600
N 0.00000000 0.00000000 -2.96995700

$HC_2\text{-}H_3Si{\cdots}NH_3$
Si 0.00000000 0.00000000 0.03941500
H 0.00000000 1.41974000 0.46555500
H 1.22953100 -0.70987000 0.46555500
H -1.22953100 -0.70987000 0.46555500
N 0.00000000 0.00000000 3.01844500
H 0.00000000 -0.94206400 3.40104800
H -0.81585200 0.47103200 3.40104800

H 0.81585200 0.47103200 3.40104800
C 0.00000000 0.00000000 -1.82310800
C 0.00000000 0.00000000 -3.03862000
H 0.00000000 0.00000000 -4.11037100

FC_2-H_3Si⋯NH_3
Si 0.00000000 0.00000000 0.76369100
H 0.00000000 1.41978100 1.18875300
H 1.22956600 -0.70989000 1.18875300
H -1.22956600 -0.70989000 1.18875300
N 0.00000000 0.00000000 3.73056200
H 0.00000000 -0.94189800 4.11358700
H -0.81570700 0.47094900 4.11358700
H 0.81570700 0.47094900 4.11358700
C 0.00000000 0.00000000 -1.09465700
C 0.00000000 0.00000000 -2.30632600
F 0.00000000 0.00000000 -3.58963600

H-F_3Si⋯NH_3
Si 0.00000000 0.00000000 0.51690100
H 0.00000000 0.00000000 1.99585900
N 0.00000000 0.00000000 -1.55725900
H -0.82434600 0.47593600 -1.91711800
H 0.00000000 -0.95187300 -1.91711800
H 0.82434600 0.47593600 -1.91711800
F 0.00000000 1.63501900 0.27480300
F -1.41596800 -0.81751000 0.27480300
F 1.41596800 -0.81751000 0.27480300

F_4Si⋯NH_3
Si 0.00000000 0.00000000 0.19456400
N 0.00000000 0.00000000 -1.83749200
H 0.00000000 -0.95188200 -2.19929100
H 0.82435400 0.47594100 -2.19929100
H -0.82435400 0.47594100 -2.19929100
F 0.00000000 1.62231400 0.01089000
F -1.40496600 -0.81115700 0.01089000
F 1.40496600 -0.81115700 0.01089000
F 0.00000000 0.00000000 1.82693300

Cl-F_3Si⋯NH_3
Si 0.00000000 0.00000000 -0.12708500
N 0.00000000 0.00000000 -2.16883700
H 0.00000000 -0.95174200 -2.53067500
H 0.82423300 0.47587100 -2.53067500
H -0.82423300 0.47587100 -2.53067500
F 0.00000000 1.62354500 -0.32887600
F -1.40603100 -0.81177300 -0.32887600
F 1.40603100 -0.81177300 -0.32887600
Cl 0.00000000 0.00000000 1.96663100

Br-F_3Si⋯NH_3
Si 0.00000000 0.00000000 -0.89621600
H 0.00000000 1.45754200 -1.15158200
H -1.26226800 -0.72877100 -1.15158200
H 1.26226800 -0.72877100 -1.15158200
N 0.00000000 0.00000000 -3.29632400
H 0.00000000 -0.94565300 -3.67175600
H 0.81895900 0.47282600 -3.67175600
H -0.81895900 0.47282600 -3.67175600
Br 0.00000000 0.00000000 1.43118000

NC-F_3Si⋯NH_3
Si 0.00000000 0.00000000 -0.10379000
N 0.00000000 0.00000000 -2.12334200

H 0.00000000 -0.95212700 -2.48530400
H 0.82456600 0.47606300 -2.48530400
H -0.82456600 0.47606300 -2.48530400
C 0.00000000 0.00000000 1.78078600
F 0.00000000 1.62626400 -0.27696300
F -1.40838600 -0.81313200 -0.27696300
F 1.40838600 -0.81313200 -0.27696300
N 0.00000000 0.00000000 2.93795100

HC_2-F_3Si···NH_3
Si 0.00000000 0.00000000 -0.09191400
N 0.00000000 0.00000000 -2.15131000
H 0.00000000 -0.95204900 -2.51095700
H 0.82449800 0.47602400 -2.51095700
H -0.82449800 0.47602400 -2.51095700
C 0.00000000 0.00000000 1.76003300
C 0.00000000 0.00000000 2.97324600
F 0.00000000 1.62710600 -0.31724400
F -1.40911500 -0.81355300 -0.31724400
F 1.40911500 -0.81355300 -0.31724400
H 0.00000000 0.00000000 4.04474000

HC_2-F_3Si···NH_3
Si 0.00000000 0.00000000 0.56483100
N 0.00000000 0.00000000 2.62444000
H 0.00000000 -0.95195900 2.98442500
H -0.82442100 0.47598000 2.98442500
H 0.82442100 0.47598000 2.98442500
C 0.00000000 0.00000000 -1.28111100
C 0.00000000 0.00000000 -2.49038800
F 0.00000000 0.00000000 -3.77256300
F 0.00000000 1.62680000 0.79074300
F 1.40885000 -0.81340000 0.79074300
F -1.40885000 -0.81340000 0.79074300
